# Central Precocious Puberty During the COVID-19 Pandemic Period: A Systematic Review of Literature

**DOI:** 10.7759/cureus.71002

**Published:** 2024-10-07

**Authors:** Maria Fanaki, Lina Michala, Ervin Nazari, George Daskalakis

**Affiliations:** 1 First Department of Obstetrics and Gynecology, ‘Alexandra’ General Hospital, National and Kapodistrian University of Athens, Athens, GRC; 2 First Surgery Clinic, Korgialeneio-Mpenakeio Hospital, Athens, GRC

**Keywords:** covid-19, early puberty, pandemic effects, pediatric endocrinology, precocious puberty

## Abstract

Central precocious puberty (CPP) is a condition where the hypothalamus-pituitary-gonadal axis is activated earlier than normal, leading to premature development of secondary sexual characteristics before eight years of age in girls and nine years of age in boys. The purpose of this study was to critically and systematically evaluate the literature regarding CPP rise during the COVID-19 pandemic.

We searched PubMed and Google Scholar for relevant articles using the following MeSH terms: "COVID-19, "precocious puberty," "early puberty," "pediatric endocrinology," and "pandemic effects." We included studies calculating the risk of CPP before and during the COVID-19 pandemic. We excluded studies looking at patients with an identifiable cause for CPP or with peripheral precocious puberty. The primary outcome was the prevalence of central precocious puberty during the pandemic compared to the pre-pandemic period. We analyzed data regarding anthropometric, biochemical, and pelvic ultrasound data between the two groups. Overall, 16 studies with 2.175 subjects were included, of which 1.818 were diagnosed with CPP. There was a rise in the number of new diagnoses of CPP during the COVID-19 pandemic (985 subjects) compared with the pre-pandemic period (833 subjects). The mean age of diagnosis in the first group was 7.42 years versus 7.54 years in the second group. Notably, CPP during the pandemic was associated with a higher body mass index (BMI) compared with the group of the pre-pandemic period (17.50 versus 17.08).

The pandemic and lockdowns led to changes in lifestyle habits, social isolation, sleep disturbance, excess screen time, and increased stress levels. We hypothesize that these alterations influenced the increase in CPP frequency.

## Introduction and background

Puberty is the result of a complex neuroendocrine process characterized by the increased release of gonadotropin-releasing hormone (GnRH) by the hypothalamus, leading to an increase in the secretion and release of gonadotropins (luteinizing hormone (LH) and follicle-stimulating hormone (FSH)) [1]. The mechanisms underlying the onset and the progression of this process have not been completely clarified, as several hormones and systems are involved [2,3]. This phenomenon is under the control of several factors, such as nutritional, genetic, and environmental factors, such as endocrine disruptors, sleep patterns, and levels of stress [4-6].

Precocious puberty (PP) is the development of secondary sexual characteristics earlier than normal, before eight years of age in girls and before nine years of age in boys [7]. There are two types of PP: central precocious puberty (CPP), which is gonadotropin-dependent PP and occurs due to early activation of the hypothalamic-pituitary-gonadal axis, and peripheral precocious puberty (PPP), which is gonadotropin-independent PP and occurs due to excess production of sex hormones from endogenous or exogenous sources [8]. Over the last decades, there has been a decline in age at puberty onset in industrialized countries; menarche appears on average in girls about 12 and a half years old, and thelarche in girls between nine and a half and ten years old [9,10]. This trend has been associated with high rates of obesity [5,11], exposure to environmental disruptors [12], and anxiety [13].

In December 2019, SARS-CoV-2 was first identified in a cluster of atypical pneumonia cases in Wuhan, China [14] and subsequently spread worldwide, causing a global pandemic. During the COVID-19 pandemic period, studies reported an increased incidence of new-onset CPP in children and acceleration of pubertal tempo of previously diagnosed patients with CPP [15,16]. The purpose of this systematic review was to critically and systematically evaluate the literature regarding CPP rise during the COVID-19 pandemic compared to the pre-pandemic period, as well as identify any relevant factors.

## Review

Materials and methods

To conduct this systematic review, a protocol based on the Preferred Reporting Items for Systematic Reviews and Meta-Analysis (PRISMA) guidelines was used, following the PRISMA assessment checklist [17]. The review is based on compiled data that has already been published in international literature. Therefore, there was no requirement for institutional review board permission or patient consent.

Eligibility Criteria, Information Sources, Search Strategy

The eligibility criteria for study inclusion were predetermined. All studies calculating the incidence of central precocious puberty during the SARS-CoV-2 pandemic period in girls in comparison to the pre-pandemic period were included in the study. Additional eligible studies were retrieved by hand-searching the citations from all articles. Reviews and studies reporting the results of surveys were excluded from the analysis. Exclusion criteria were also studies that were not written in English, studies including patients with an identifiable organic cause for central precocious puberty or with peripheral precocious puberty, and studies calculating the incidence of CPP in boys. All studies meeting the inclusion criteria were included in the review. Information regarding the publication date, the main findings, and the number of patients and controls was recorded for every eligible article. A literature review of the available publications retrieved by a search in various scientific databases, including PubMed and Google Scholar, was performed using the following MeSH terms: [(Covid-19 or pandemic effects) and (precocious puberty or early puberty or pediatric endocrinology)].

**Figure 1:**
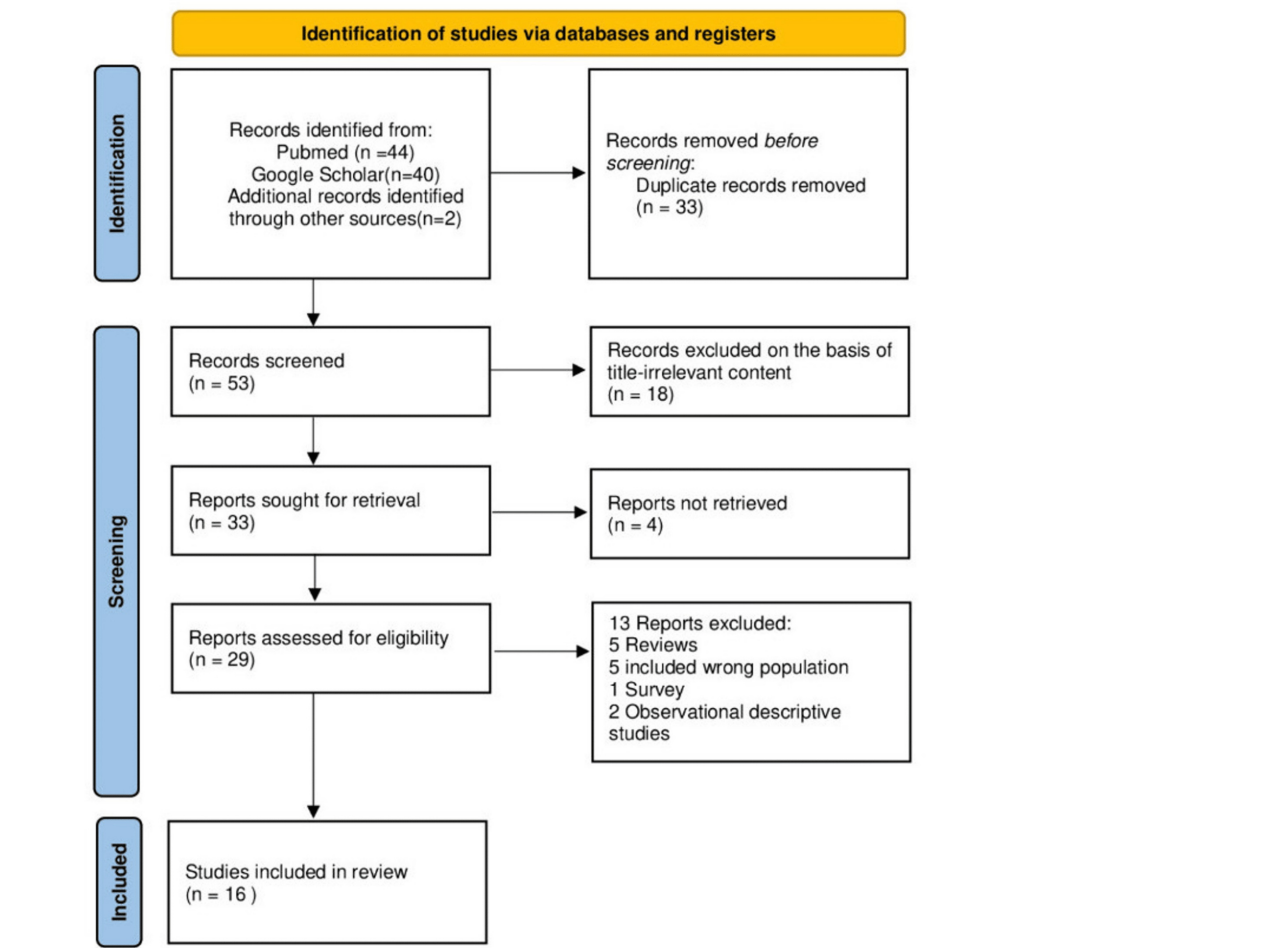
Prisma flowchart PRISMA: Preferred Reporting Items for Systematic Reviews and Meta-Analysis

The available data were published from February 2020 to December 2023, and our last search was set for January 7, 2024. The PRISMA flow diagram schematically presents the stages of article selection (Figure 1).

Study Selection

Three phases of selection were involved in the retrieval of research. After removing duplicate articles, two authors (MF and EN) manually reviewed the titles and abstracts of the remaining electronic articles to assess their eligibility. Any disagreements were resolved through discussion with LM and GD. Finally, studies deemed potentially eligible were selected for inclusion after the full text was retrieved and reviewed. Any disagreements at this stage were resolved through consensus among all authors.

Data Extraction

The outcome measures were established in advance during the planning phase of this systematic review. The primary outcome of our study was to identify the prevalence of central precocious puberty during the pandemic compared to the pre-pandemic period. Anthropometric, biochemical, and pelvic ultrasonography data were compared between the two groups as secondary outcomes.

Assessment of Risk of Bias

The methodological quality of the included observational studies was assessed by two authors (MF and EN) using the Newcastle-Ottawa Scale (NOS) score (Table 1). This scale evaluates the risk of bias in observational studies by assessing the selection of study groups (maximum four points), the comparability of the groups (maximum two points: one point for comparable age and one point for comparable basal LH values), and the outcome of interest, which was predefined as the incidence of central precocious puberty: the early onset of secondary sexual characteristics in girls before the age of eight (maximum three points) [13,18-33].

**Table 1:** Newcastle-Ottawa Scale

Study	Selection				Comparability	Outcome		
	Adequacy of case definition	Representativeness of cases	Selection of controls	Definition of controls	Comparability (Age/baseline LH)	Assessment of outcome	Adequate follow-up period	
Acinikli et al., 2022 [18]	√	√	√	√	√√	√	√	
Baby et al., 2023 [19]	√	√	√	√	√√	√	√	
Barberi et al., 2022 [20]	√	√	√	√	√√	√	√	
Orman et al., 2022 [21]	√	√	√	√	√√	√	√	
Chioma et al., 2021 [22]	√	√	√	√	√√	√	√	
Fava et al., 2023 [23]	√	√	√	√	√√	√	√	
Fu et al., 2022 [24]	√	√	√	√	√√	√	√	
Goggi et al., 2023 [25]	√	√	√	√	√√	√	√	
Itani et al., 2022 [26]	√	√	√	√	√√	√	√	
Matsubara et al., 2022 [27]	√	√	-	√	√√	√	√	
Mutlu et al., 2022 [28]	√	√	-	√	√√	√	√	
Oliveira Neto et al., 2022 [29]	√	√	√	√	√√	√	√	
Stagi et al., 2022 [30]	√	√	√	√	√√	√	√	
Umano et al., 2020 [31]	√	√	√	√	√√	√	√	
Verzani et al., 2022 [32]	√	√	√	√	√√	√	√	
Frank et al., 2021 [33]	√	√	√	√	√-	√	√	

Results

A total of 86 articles were found across all databases using the search strategy, with 33 being duplicates. Based on our inclusion and exclusion criteria, 16 studies were deemed eligible and included in this systematic review. Table 2 presents the study characteristics of included studies, while Table 3 provides a cumulative report comparing the characteristics of the patients with CPP during the pandemic period to the pre-pandemic period.

**Table 2:** Study characteristics CPP: central precocious puberty; BMI: body mass index; FSH: follicle-stimulating hormone; LH: luteinizing hormone; GnRH: gonadotropin-releasing hormone

Study and Year	Patients (n)	Study type	Duration of study	Inclusion criteria	Exclusion criteria
Acinikli et al., 2022 [18]	89	Retrospective	March 2020 to June 2021	Girls diagnosed with CPP within the 15 months before and the 15 months following the beginning of the COVID-19 pandemic. CPP was defined when breast development was evident before the age of 8 years with a baseline (LH) value of >0.3 mIU/mL and/or a stimulated LH value of >5 mIU/mL.	Cases involving peripheral precocious puberty, premature thelarche, premature adrenarche, and male patients were excluded.
Baby et al., 2023 [19]	56	Retrospective	March 2020 to March 2021	Girls, 5-8 years old, who were seen for puberty-related concerns from March 2018-March 2021 at the Endocrine Clinic in the Fink Ambulatory Care Center in New York University Langone Medical Center. CPP was defined when the breast Tanner stage was greater than 1 with either elevated basal LH (0.3mIU/L) and/or estradiol ≥36.0 pg/m, and/or ovarian volumes >1 cm3.	Girls who were adopted or had hypothalamic-pituitary congenital malformations, neurological, neurosurgical, genetic, oncological diseases, or peripheral precocious puberty were not included.
Barberi et al., 2022 [20]	26	Retrospective	March 2020 to April 2021	Girls were observed in two periods: Period 1, before lockdown (1 January 2019 – 8 March 2020), and Period 2, lockdown and the following months (9 March 2020 – 30 April 2021).	Girls with hypothalamic-pituitary congenital malformations, neurological, neurosurgical, and/or genetic diseases, psychomotor delay, oncological diseases, and adopted girls were excluded.
Orman et al., 2022 [21]	400	Cross-sectional	2016 to 2022	Girls ≤6 years referred from March to August 2016-2020 were included in the study. CPP was defined when breast development was greater than Tanner stages ≥B2 before the chronological age of 8 years.	Patients with abnormal brain and pituitary gland MRI reports, including Rathke’s cleft cysts, pineal cysts, arachnoid cysts, hamartoma, and germinoma; other endocrine diseases, including hypothyroidism, hyperthyroidism, short stature, diabetes and adrenal disease; and chronic diseases, including chronic nephrosis, asthma, epilepsy, and hematological disease were excluded.
Chioma et al., 2021 [22]	468	Retrospective	March 2019 to September 2020	Patients were observed in five Italian tertiary centers of Pediatric Endocrinology, in the periods March–September 2020 and March–September 2019. CPP was defined when a detectable basal LH level (>0.2 IU/L) and/or a peak response of LH after GnRH infusion >5 IU/L, and/or serum estradiol levels >20 pg/mL.	Girls ≤3 years, susceptible to developing precocious puberty, and those who were lost during follow-up were excluded.
Fava et al., 2023 [23]	133	Retrospective	January 2016 to June 2021	Girls with CPP diagnosed between January 2016 and February 2020 were separated into two groups: Group 1 before the COVID-19 pandemic, and Group 2 during the pandemic from March 2020 until the end of June 2021. CPP was defined as breast development Tanner stage ≥2 before 8 years of age, and 1 or more of the following criteria: height velocity (HV) >6 cm/years, advanced bone age by at least 1 year, basal serum LH >0.3 U/L, peak LH >5 U/L after LH releasing hormone test, and negative brain magnetic resonance imaging (MRI).	Girls with premature thelarche, those with a genetic syndrome, brain tumor, or other preexisting condition, and those with peripheral precocious puberty or with isolated premature thelarche were excluded.
Fu et al., 2022 [24]	182	Retrospective	February 2018 to May 2020	Girls aged 5–9 years between February and May 2020 and diagnosed with CPP and PT according to the respective diagnostic criteria, residents in Henan Province for >3 years, willing to cooperate with questionnaire surveys, and related examinations after informed consent were included.	Girls with CPP caused by intracranial lesions, unwilling to cooperate with questionnaire surveys or clinical auxiliary examinations, with a previous diagnosis of PP, regardless of treatment, and with PPP with a clear cause, such as exogenous drug abuse were excluded.
Goggi et al., 2023 [25]	49	Retrospective	2014-2021	Girls with CPP were divided into two groups: Group 1 (Post-lockdown Group) including patients whose first signs of CPP appeared as of the beginning of lockdown in March 2020 until July 2021; Group 2 (Pre-lockdown Group) comprising patients whose first signs of CPP appeared between 2014 and February 2020. CPP is defined as thelarche before the age of 8 for females, LH levels of 5 mU/L or greater (either basal or after stimulation with GnRH) detected within the age of 9.	Patients who were male or who had a recognized cause (such as CNS lesions or genetic variations known to be harmful) for their central precocious puberty were not included in the analysis.
Itani et al., 2022 [26]	23	Retrospective	March 2020 to February 2021	Lebanese girls only, aged between 4 and 9 years old. Precocious puberty was defined as an association between pubertal hormonal and radiological findings (LH values of >5 IU/L on the LHRH stimulation test, a ratio of stimulated LH to stimulated FSH of >1.0, or a basal LH value of >0.1 IU/L, estradiol > 10pg/mL with uterus size more than 34 cm) and a clinical onset of puberty (Tanner stage 2, breast development isolated or associated with pubic and/or axillary hair with acceleration in the growth velocity) at an age less than 8 years.	Exclusions from the study were individuals taking medications that could affect pubertal development, patients with psychomotor and developmental delay, patients with hypothalamic-pituitary congenital abnormalities, and patients with endocrine impairment requiring prior hormonal treatment.
Matsubara et al., 2022 [27]	79	Retrospective	April 2019 to April 2021	Girls between the ages of three and thirteen were selected, and they were divided into two groups: the "Before COVID-19 pandemic" group, which included CPP cases from April 7, 2019 to April 6, 2020, and the "After COVID-19 pandemic" group, which included CPP cases from April 7, 2020 to April 6, 2021. CPP was defined as breast development at <7 yr and 6 mo of age, pubic hair development, labia minora pigmentation or axillary hair development at < 8yr of age, and menarche at <10 yr and 6 mo of age; basal LH value ≥0.5 mIU/mL or peak LH value ≥5.0 mIU/mL at the time of GnRH stimulation test.	Patients with Graves' illness, small-for-gestational-age short stature, chromosomal abnormalities, congenital hypothalamic-pituitary malformations, intracranial space-occupying lesions, severe motor and intellectual deficits, and those undergoing medication treatment were not included.
Mutlu et al., 2022 [28]	61	Retrospective	March 2019 to September 2020	Cases divided into two groups: the pre-pandemic group consisted of girls referred between March 9, 2019, and March 9, 2020, and the pandemic group between March 9, 2020, and September 9, 2020. Precocious puberty was defined as the breast development with or without axillary/pubic hair before the age of 8.; a baseline LH level of >1.1 IU/L together with pubertal signs, or a GnRH-stimulated peak LH level of >5 IU/L with a stimulated LH/FSH ratio of >1.0.	Cases with non-idiopathic central precocious puberty (CPP), isolated premature adrenarche, and peripheral precocious puberty were excluded.
Oliveira Neto et al., 2022 [29]	55	Cross-sectional	March 2019 to June 2021	Cases are divided into two groups: the pandemic group, from July 2020 to June 2021, and the pre-pandemic group from March 2019 to February 2020. Precocious puberty was defined as the development of pubertal changes at age less than 8 years in girls in the presence of LH >0.3 IU/L, LH peak >5 IU/L on GnRH stimulation test, or ovarian volume >2 cm3 at pelvic ultrasound.	Patients with precocious puberty linked to hypothalamic-pituitary malformations, neurological, neurosurgical, or genetic conditions, psychomotor retardation, cancer, or other endocrine disorders requiring hormonal treatment or affecting pubertal development were excluded.
Stagi et al., 2022 [30]	54	Retrospective	April 2019 to April 2020	Patients who were diagnosed with CPP and started GnRH analog therapy between April 1 to July 1, 2019 (Group 1), and April 1 to July 1, 2020 (Group 2). Precocious puberty diagnostic criteria are that breast development starts before the age of 8 in girls, basal LH value is above 0.3 IU/L, and GnRH stimulation test LH peak was over 5 IU/L.	Patients with known endocrinological problems and patients with organic lesions on cranial magnetic resonance imaging were not included.
Umano et al., 2020 [31]	126	Retrospective	March 2015 to July 2020	Cases divided into two groups: Group 1- during the lockdown from March to July 2020, and Control Group- from March 2015 to July 2015. Precocious puberty was defined as the development of pubertal changes before the age of 8; LH values of >5 IU/L on the GnRH in the presence of pubertal signs or a basal LH value of >1.1 IU/L and a ratio of stimulated LH to stimulated FSH of >1.0 combined with isolated and/or axillary hair growth accompanied by breast development.	Patients were excluded if they had hypothalamic-pituitary congenital malformations, neurological, neurosurgical, or genetic diseases, psychomotor delay, cancer, other endocrine disorders needing hormonal treatments, were taking drugs that could interfere with pubertal development, or were adopted or immigrants.
Verzani et al., 2022 [32]	72	Prospective	2017 to 2020	Cases with CPP were divided into two groups: Group 1 during the lockdown period from April 2020 to April 2021, and Group 2- from 2017 to 2020. CPP was defined as breast development before 8 years of age and basal luteinizing hormone (LH) levels (LH >0.3 UI/L) and/or LH levels >5 IU/L on the GnRH-stimulated test.	Not mentioned.
Frank et al., 2021 [33]	302	Retrospective	2019 to September 2020	Cases divided into two groups: Group 1 during the pandemic from March 2020 to September, and Group 2 /pre-pandemic- from March 2019 to September 2019. The definition of CPP was not mentioned.	Not mentioned.

**Table 3:** CPP during the pandemic period versus CPP pre-pandemic period CPP: central precocious puberty; BMI: body mass index; FSH: follicle-stimulating hormone; LH: luteinizing hormone

Study and Year	Age (y)	Weight (kg)	Height (cm)	BMI (kg/m2)	Bone age (y)	Basal FSH (mIU/ml)	Basal LH (mLU/ml)	Estradiol (pg/ml)	Ovarian volume (cc)
Acinikli et al., 2022 [18]	7.5/ 7.5	NM/ NM	NM/ NM	NM/ NM	8.8/ 8.2	3.7/ 3.3	0.5/ 0.7	18/ 19	NM/ NM
Baby et al., 2023 [19]	7.1/ 6.9	26.9/ 26.8	126/ 124.5	16.8/ 17.3	NM/ NM	2. 9/ 2.4	1.7/ 1	21.9/ 9.7	2,8/ 1.8
Barberi et al., 2022 [20]	6.43/ 6.96	NM/ NM	NM/ NM	NM/ NM	NM/ NM	NM/ NM	1.15/ 2.5	NM/ NM	NM/ NM
Orman et al., 2022 [21]	7.95/ 7.92	NM/ NM	NM/ NM	17.48/ 17.12	NM/ NM	2.77/ 3.6	0.72/ 0.67	21.35/ 22.31	NM/ NM
Chioma et al., 2021 [22]	7.04/ 7.04	NM/ NM	NM/ NM	NM/ NM	9.95/ 9.18	3.95/ 3.4	1.21/ 1.33	12.13/ 9.05	NM/ NM
Fava et al., 2023 [23]	6.89/ 7.03	NM/ NM	NM/ NM	NM/ NM	7.55/ 9.75	3.69/ 4.3	1.26/ 1.75	20.97/ 19.49	2.20/ 2.51
Fu et al., 2022 [24]	7.31/ 7.21	32.93/ 22.77	132.76/ 123.32	18.02/ 15.07	8.91/ 7.13	3.48/ 1.7	1.96/ 0.11	30.12/ 11.72	NM/ NM
Goggi et al., 2023 [25]	7.43/ 7.22	27.3/ 29.35	130.25/ 129.03	17.49/ 17.62	9.43/ 8.8	3.8/ 3.8	0.6/ 0.6	22.88/ 10.35	1.92/ NM
Itani et al., 2022 [26]	7.27/ 6.66	28.67/ 21.2	128/ 119.6	17.37/ 14.85	8.52/ 6.87	3.45/2	0.93/0.11	27.1/ 15	2.66/ 0.64
Matsubara et al., 2022 [27]	8,5/ 9	NM/ NM	NM/ NM	NM/ NM	10,3/ 11	4.6/ 3.7	1.6/1.9	22.9/ 21.4	NM/ NM
Mutlu et al., 2022 [28]	7.7/ 7,9	NM/ NM	NM/ NM	NM/ NM	9.14/ 9.4	NM/ NM	0.78/ 0.74	16.5/ 19.5	2.4/ 3
Oliveira Neto et al., 2022 [29]	7.7/ 7.86	NM/ NM	NM/ NM	NM/ NM	9.55/ 9.82	4.33/ 4.1	1.87/ 1.35	25.61/ 27.44	1.88/ 3.15
Stagi et al., 2022 [30]	7.92/ 8.54	30.4/ 36.1	123.82/ 135.76	17.97/ 19.47	8.78/ 9.78	2.94/ 3.3	0.61/ 0.38	16.54/ 13.95	NM/ NM
Umano et al., 2020 [31]	7.11/ 7.53	NM/ NM	NM/ NM	NM/ NM	9.40/ 9.60	1.9/ 2.2	1.2/ 1.9	35.38/ 32.03	3.32/ 2.83
Verzani et al., 2022 [32]	7.59/ 7.97	NM/ NM	129.69/ 129.25	NM/ NM	8.77/ 9.33	7.09/ 4.3	1.23/ 0.87	30.88/ 19.38	NM/ NM
Frank et al., 2021 [33]	7.33/ 7.51	28.27/ 30.67	126.96/ 129.06	17.39/ 18.19	NM/ NM	NM/ NM	NM/ NM	NM/ NM	NM/ NM

Overall, 2.175 subjects were included, 1.818 of them newly diagnosed cases with CPP. The subjects were divided into two groups according to the time of diagnosis. We reported 985 newly diagnosed cases of CPP during the pandemic period, with a mean age of 7.42 years (range: 6.43 to 8.5 years). On the contrary, we accumulated 833 new diagnoses of CPP in the pre-pandemic period, with a mean age of diagnosis of 7.54 years (range: 6.66 to 9 years). Notably, CPP in the pandemic was associated with a higher body mass index (BMI) compared with the group of the pre-pandemic period (17.50 versus 17.08). The mean bone age of the first group was 9.09 years, whereas the mean age of the second group was 9.07 years. In particular, the mean ovarian volume in the group of cases during the pandemic period was lower compared to the group of cases in the pre-pandemic period. Mean basal FSH, LH, and estradiol in the cases diagnosed during the SARS-CoV2 period were 3.73 mIU/ml, 1.15 mIU/ml, and 23.01 pg/ml in regard, whereas in the group of cases before the pandemic were significantly lower (3.08 mIU/ml, 1 mIU/ml, and 15 pg/ml).

Discussion

Puberty can be defined as the transition period between childhood and adolescence. Reproductive development is controlled by the hypothalamic-pituitary axis (HPA). One of the main hormones in controlling the reproductive system is gonadotropin-releasing hormone (GnRH). It is produced in periodic bursts rather than a continuous stream because the hypothalamus releases it in a pulsatile way. This pulsatile secretion influences the release of two other hormones from the anterior pituitary gland: luteinizing hormone (LH) and follicle-stimulating hormone (FSH), and it is essential for the proper functioning of the reproductive axis. After the on-off neurosecretion of GnRH, LH, and FSH levels increase and enhance the production of sex hormones by the gonads, causing alterations in secondary sexual characteristics and affecting the growth rate, bone mineralization, and the function of the central nervous system [33].

In this systematic review, we identified an elevation of CPP prevalence during the COVID-19 pandemic period compared with before the pandemic period. The findings suggest a complex interplay between environmental, psychological, and possibly viral factors contributing to an increased incidence of precocious puberty during the COVID-19 pandemic. Studies from different centers reported that the number of patients with PP increased from 86,352 cases in 2016 to 166,645 cases in 2021 in Korea [34]. According to Geniuk et al., the frequency of patients with suspected PP increased 2.3-fold during the pandemic period [35]. The exact reason for the rise of cases of precocious puberty during quarantine is not well established, but several possible mechanisms have been proposed. Due to the high transmission rate of COVID-19, several countries imposed restrictive policies promoting social distancing and quarantine measures to reduce the spread, the deaths, and the healthcare costs caused by SARS-CoV-2 [36]. These measures affected many aspects of life, leading to a radical change in habits and family lifestyle. While the children were forced to stay at home, they became less physically active, spent more time in front of screens, experienced sleep disturbances, and followed an unhealthy diet [37-39]. According to Bruni et al. [40], children across all age groups had a markedly altered sleep/wake cycle, with a large proportion of them indicating a notable delay in going to bed and getting up during the pandemic period. In addition, in a cross-sectional study involving 1,342 participants, it was discovered that more people were using digital media right before bed, staying up later, and spending more time in bed. Surprisingly, however, these individuals, especially those with higher stress and anxiety levels, also have lower quality of sleep [41]. These drastic changes could have acted as triggers for the early initiation of puberty.

Several reports showed that CPP could be related to higher BMI and obesity in children [42-44]. During the pandemic, lockdowns, sedentary lifestyles, and more frequent consumption of fast food led to rapid growth in children, which is often associated with excess weight. In particular, the results of a retrospective survey demonstrated that during the COVID-19 lockdown, the mean body mass index of all participating youths increased significantly (21.8-22.6), as did the prevalence of obesity (10.5% to 12.9%, P <.001) and overweight/obesity (21.3%-25.1%, P <.001). Additionally, there had been a notable shift in their patterns of activity, which included less frequent participation in active modes of transport, moderate-to-vigorous housework, walking during leisure time, and an increase in screen time, sedentary behavior, and sleeping [45]. Another study reported that during the pandemic consumption of potato chips, red meat, and sugary drinks increased dramatically (P-value range, 0.005 to<0.001) [46]. Obese children have a lower concentration of adipokines (particularly leptin and adiponectin) that may suppress the secretion of kisseptin and GnRH by hypothalamic neurons and play an important role in the onset of puberty [47].

The excess use of digital devices during the COVID-19 pandemic may also be correlated to precocious puberty. A survey including 11,391 participants revealed a significant increase in screen use; 4125/11,391 (36.21%) of the sample indicated higher screen use, and 1729 (15.18%) reported having difficulties in managing their screen use [48]. Many recent studies have reported that exposure to electromagnetic fields, including smartphones and tablets, inhibits melatonin production [49,50]. Normally, nocturnal serum melatonin levels are high during childhood and gradually decrease during adolescence. Low melatonin levels influence the hypothalamic-pituitary-gonadal (HPG) axis, therefore causing rapid pubertal maturation. Furthermore, more screen usage could lead children to be more susceptible to depression, anxiety, suicide, and inattentiveness [51].

Endocrine-disrupting chemicals could be another contributing factor, particularly as hygiene precautions caused an increase in the use of disposable items such as polybrominated flame retardants, phthalate esters, and bisphenol A that are known to be factors stimulating puberty as they act as estrogen agonists and/or testosterone antagonists [52-54].

Stress impacts the HPA axis and may alter pubertal timing, a theory supported by the significant psychological burden observed during the pandemic. Knight et al. reported that anxiety in pre-pubertal girls is associated with early pubertal initiation [13]. Certainly, children experienced social distancing and less physical activity as a stressful condition [55,56]. Moreover, the fear of illness, as well as financial concerns, and the high rate of domestic violence during quarantine could also be stress triggers for children [57]. Animal studies reported that increased catecholamines (dopamine and norepinephrine (NE)) trigger the initiation of puberty [58]. Methylphenidate, a drug used for the treatment of attention deficit hyperactivity disorder, which inhibits dopamine and NE transporters and increases their concentration in synaptic gaps, has been related to precocious puberty [59].

Besides the secondary effects of social distancing and isolation, the SARS-CoV-2 pandemic may have had effects per se on pubertal development through direct modifications in the central nervous system. The SARS-CoV-2 virus binds to the angiotensin-converting enzyme-2 receptor in the cranial nerve system, especially around the olfactory bulb, where there is a high concentration of GnRH neurons and gamma-aminobutyric acid (GABA)ergic neurons. The SARS-CoV-2 may also accelerate puberty onset due to the disruption of the blood-brain barrier and direct interaction with the neural system [60]. The COVID-19 infection causes an inflammatory cytokines storm that could also activate N-methyl-D-aspartate (NMDA) receptors, stimulating GnRH pulsatile secretion through inputs from neurotransmitters such as glutamate [61].

## Conclusions

This review highlights the multifaceted influences of pandemic-related lifestyle changes, stress, and potential direct effects of SARS-CoV-2 infection. Ongoing research is crucial to fully understand the implications and to guide future clinical practice. Further research is needed to understand long-term impacts and develop intervention strategies to mitigate adverse outcomes associated with early puberty.
